# Supplementary material for: Ceiling effect of clomiphene citrate on the testosterone to estradiol ratio in eugonadal infertile men
Source: PLoS One. 2022 Jan 31;17(1):e0262924. doi: 10.1371/journal.pone.0262924 (PMC8803167; doi:10.1371/journal.pone.0262924)
Supplement: Attachment — Submitted filename: Response to Reviewers.docx [file pone.0262924.s001.docx]

Reviewer #1: 
**Q:** 1. As aromatase breaks down T to E2, would report on the effect of BMI on results/outcomes.

**Answer:** Different BMI levels may probably affect T/E2 ratio. However, we did not include patients’ BMI in our data base. Your suggestion is good for further study of this topic and data of BMI would make a more precise analysis.

**Q:** 2. Should the Cirrhosis patient be excluded since liver disease effects sex hormone binding globulin production (effects total Testosterone)?

**Answer:** Cirrhosis surely affect sex hormone binding globulin production, indirectly influencing the bioavailability of testosterone. As for the patient included in our study with liver cirrhosis, his Child-Pugh score is only grade A with normal bilirubin, PT/APTT and albumin level, which may hardly have an impact on sex hormone binding globulin production.

**Q:** 3. Is the standard to use ng/mL for T? I'm more used to ng/dL...with the normal T:E ratio being 10:1.

**Answer:** There may not be a standard unit of testosterone level. According to EAU(European Association of Urology) guideline, ng/mL and nmol/L have both been used as the unit of testosterone level.

Reviewer #2: 
**1) Introduction:**

**Q:** I did not observe references for all the information contained in the paragraph below. Despite being general domain information, it is important to always add reference sources, even if it is in the introduction.
“Selective estrogen receptor modulators (SERMs) are drugs that act on estrogen receptors. Such drugs can be divided into receptor agonists and antagonists (Ref?). These drugs selectively act on specific organs, with effects differing by organs (Ref?). Clomiphene citrate is both a nonsteroidal antiestrogen drug and an SERM that competes with estradiol for estrogen receptors in the hypothalamus and blocks the normal negative feedback of circulating estradiol on the hypothalamus (Ref?). The production of gonadotroptin-releasing hormone (GnRH) may not be limited by estrogen; instead, the pituitary gland releases more follicle-stimulating hormone (FSH) and luteinizing hormone (LH) and causes an increase in sperm and testosterone production by the testes (Ref?). High levels of intratesticular testosterone is a key factor in the nonconventional pathway of testosterone for spermatogenesis (Ref?).

**Answer:** We really appreciate the advice of adding references. Three references have been added to the introduction. The following paragraph is the revised introduction.

“Selective estrogen receptor modulators (SERMs) are drugs that act on estrogen receptors and such drugs can be divided into receptor agonists and antagonists [1]. These drugs selectively act on specific organs, with effects differing by organs [1]. Clomiphene citrate is both a nonsteroidal antiestrogen drug and an SERM that competes with estradiol for estrogen receptors in the hypothalamus and blocks the normal negative feedback of circulating estradiol on the hypothalamus [2]. The production of gonadotroptin-releasing hormone (GnRH) may not be limited by estrogen; instead, the pituitary gland releases more follicle-stimulating hormone (FSH) and luteinizing hormone (LH) and causes an increase in sperm and testosterone production by the testes [2]. High levels of intratesticular testosterone is a key factor in the nonconventional pathway of testosterone for spermatogenesis [3].”

Reference:

1. Chua, M.E., et al. Revisiting oestrogen antagonists (clomiphene or tamoxifen) as medical empiric therapy for idiopathic male infertility: a meta-analysis. Andrology, 2013. 1: 749.
2. Ribeiro, R.S., et al. Clomiphene fails to revert hypogonadism in most male patients with conventionally treated nonfunctioning pituitary adenomas. Arq Bras Endocrinol Metabol, 2011. 55: 266.
3. Hussein, A., et al. Clomiphene administration for cases of nonobstructive azoospermia: a multicenter study. J Androl, 2005. 26: 787.

**2) Materials and Methods**
**Q:** I would like the authors to specify which reference was used to define the diagnosis of eugonadism (total testosterone 2.4 to 8.7 ng/mL).
The laboratory diagnosis of hypogonadism is based on the measurement of total testosterone. The American Society of Endocrinology recommends the value of 264 ng/dL (9.2 nmol/L) as the lower limit to define the hypogonadal state; however, the cutoff levels for diagnosis are not a consensus. So I would like the authors to specify the Guideline used.
(Testosterone Therapy in Men With Hypogonadism: An Endocrine Society* Clinical Practice Guideline)

**Answer:** Exactly I agree that the cutoff level of hypogonadism and eugonadism has not reached a consensus. Different hospital may have different lab reference level with regard to their laboratory department. All patient included in our study have done blood examination in National Taiwan University Hospital. The reference range of total testosterone(2.4 to 8.7 ng/mL) is defined by [Department of Laboratory Medicine](https://www.ntuh.gov.tw/labmed/Index.action) of National Taiwan University Hospital.

**Q:** I would like the authors to write “range of the total testosterone level” instead of just “range of the testosterone level” – here it can be confusing if total or free testosterone was used as a parameter.

**Answer:** We really appreciate the advice of modifying “testosterone” to “total testosterone” not to be confusing. Adjustment have been made throughout the whole manuscript(changing the testosterone level to the total testosterone level)

**Q:** I would like more detail in the table of 22 patients at baseline with data about BMI, waistline and testicular volume if possible

**Answer:** BMI level, waistline and testicular volume are surely the possible factor that may affect total testosterone and estradiol level, indirectly influencing the T/E2 ratio. Unfortunately, these parameters were not included in our study. Due to the retrospective nature of our study, these factors were not recorded in our data base. Further prospective study including these factors may be needed.

**3) Discussion**
**Q:** An important point to be considered is the ability of the CC to induce a response in patients with central or mixed hypogonadism. In the different studies in which its use was evaluated, it was possible to define response (elevation of testosterone) in patients with Leydig cells capable of reacting to the central stimulus restored by CC (that's why I would like to know about testicular volume).
It is known that the unbalance of the T:E2 ratio is directly implicated in the pathophysiology of hypogonadism. It is important to emphasize that the increase in estrogen with the use of CC results from the aromatization of testosterone in the adipose tissue, especially the visceral tissue. I would like to see the details of the abdominal fat of these patients in order to understand whether the ceiling effect proposed by the authors is related to patients with larger abdominal fat/waistline and therefore greater visceral fat.

**Answer:** According to EAU (European Association of Urology) guideline, larger abdominal fat and greater visceral fat has now been established to be related to hypogonadism. Increase in estrogen by aromatase in visceral fat has also been established in several studies. Unfortunately, these factors were also not included in our study and may not be achieved due to the retrospective nature of our study. We will work our best on the further prospective study of relevant topics.
