# Supplementary material for: Ceiling effect of clomiphene citrate on the testosterone to estradiol ratio in eugonadal infertile men
Source: PLoS One. 2022 Jan 31;17(1):e0262924. doi: 10.1371/journal.pone.0262924 (PMC8803167; doi:10.1371/journal.pone.0262924)
Supplement: Attachment — Submitted filename: Response to Reviewers.docx [file pone.0262924.s002.docx]

Reviewer #2: 

Q: 1) In this part of the manuscript, I would like to correct “A small population study by Shabsigh [5] also reported an increased T/E ratio after clomiphene use in patients with hypogonadism” to “A small population study by Shabsigh [5] also reported an increased T/E2 ratio after clomiphene use in patients with hypogonadism”. The number 2 on E was missing.

A: We really appreciate for checking through the manuscript for any missing word or number. “Number 2” has now been added to T/E ratio.

Q: 2) I would like the description of the laboratory kits used for the analysis of total testosterone level, estradiol level, LH, FSH and prolactin level to be detailed.

A: We add the following description about the measurement methods in my document. All hormone test(testosterone, estradiol, LH, FSH and prolactin) included in our study are sent to the central lab in “[Department of Laboratory Medicine](https://www.ntuh.gov.tw/labmed/Index.action) of National Taiwan University Hospital”. FSH, LH, E2 and prolactin were measured by using IMMULITE^®^ 2000 immunoassay system and Testosterone was measured by the ARCHITECT^®^ 2nd Generation Testosterone assay which is a chemiluminescent microparticle immunoassay for the quantitative determination of testosterone.

Q:3) I would like to know if the dosage of sex hormone binding globulin (SHBG) levels was measured via morning lab draws.

A: Sex hormone binding globulin (SHBG) levels is surely a possible factor that may affect bioavailable testosterone, indirectly influencing the T/E2 ratio. Unfortunately, this parameter was not included in our study. Due to the retrospective nature of our study, this factor was not recorded in our data base. Further prospective study including this factor may be needed.

Q:4) To make it easy in terms of standardization, I would like to put the total testosterone level in ng/dL and estradiol levels in ng/dL (<https://doi.org/10.1016/j.juro.2017.02.2652>)

A: We really appreciate for this suggestion. All testosterone level and estradiol level have been changed to ng/dL. T/E2 ratio has also been adjusted due to the unit change of testosterone and estradiol.

Q:5) In the “limitations” part of the study, I would like it to be detailed that there wasn't possible to get BMI level, waistline and testicular volume data. I suggest that the authors should proceed with the study to evaluate obese men versus men with normal weight and analyse if there are differences in the ceiling effect.

A: We appreciate for the advice of adding this part to out limitation. We surely will proceed with the study to include these parameters for analysis to see if there are differences in the ceiling effect.

Q:6) If I may suggest, a greater number of participants would improve the strength of the results

A: Thanks for reviewer’s nice suggestion. Small study population is surely a limitation of our study. Further studies will include more participants to strengthen our result.

Q:7) The study generates an interesting hypothesis about which subgroup of patients may benefit most from the use of clomiphene citrate

A: We are glad that this study leads to an interesting result of “Clomiphene citrate significantly increase the T/E2 ratio in eugonadal infertile men under the premise of its ceiling effect (T/E2 ratio < 200)”. We would work hard on increasing the sample size and including more important parameters for further analysis.
